# Radiomics-based identification of benign and malignant orbital lesions using contrast-enhanced CT imaging

**DOI:** 10.1097/MD.0000000000045791

**Published:** 2025-11-07

**Authors:** Weitao Huang, Xiaowei Han, Guozheng Zhang, Xiaohui Liu

**Affiliations:** a Department of Radiology, The Quzhou Affiliated Hospital of Wenzhou Medical University, Quzhou People’s Hospital, Quzhou, China.

**Keywords:** benign, computed tomography, differential diagnosis, malignant, orbit, radiomics

## Abstract

This study aimed to evaluate the value of contrast-enhanced computed tomography (CT) imaging radiomics in distinguishing malignant lesions from benign ones in the orbit. A retrospective analysis was conducted on CT imaging data from 139 patients with orbital tumor lesions, all of whom underwent contrast-enhanced CT scans within 2 weeks before diagnosis. Of these, 45 cases were benign lesions and 94 were malignant lesions. Radiomic features were extracted from the contrast-enhanced CT images, and 12 features were selected through the minimum redundancy maximum relevance and least absolute shrinkage and selection operator regression methods. The selected features were used to build models using logistic regression, Naive Bayes Classifier (NaiveBayes), support vector machine (SVM), Extra Trees Classifier (ExtraTrees), and multilayer perceptron, with the best-performing model identified. Multivariate logistic regression was employed to identify clinical risk factors for malignant orbital lesions, and a nomogram model was developed by combining radiomic features and clinical variables. The predictive performance of each model was evaluated using the area under the receiver operating characteristic curve. Among the 3 machine learning models, the SVM model demonstrated the best predictive performance and robustness across datasets. Therefore, the SVM model was used to construct the nomogram. The nomogram achieved area under the receiver operating characteristic curve values of 0.957 and 0.833 in the training and testing cohorts, respectively, both of which were higher than 0.80. The performance of the nomogram was significantly superior to that of the clinical model (De-long test, *P* < .05), but no statistically significant difference was observed when compared to the radiomics model (De-long test, *P* > .05). Contrast-enhanced CT radiomics can effectively differentiate between malignant and benign orbital lesions. Both the nomogram and radiomics models exhibited high predictive performance, offering valuable insights for clinical decision-making.

## 1. Introduction

Orbital lesions are a common clinical condition encompassing a wide range from benign to malignant entities. These lesions include orbital tumors, infectious lesions, inflammation, and vascular malformations, which can affect vision, ocular motility, and facial appearance.^[1]^ Due to the complex anatomical structure of the orbit, and the proximity of lesions to surrounding tissues, radiological diagnosis requires comprehensive consideration of factors such as lesion location, morphology, size, and other characteristics. However, due to the diversity of orbital lesions and their varied clinical manifestations, traditional imaging methods like X-rays, computed tomography (CT), and magnetic resonance imaging (MRI), although providing valuable structural information, may fail to accurately distinguish between benign and malignant orbital lesions in some cases, especially when lesions are in the early stage or when tumor boundaries are unclear.^[2,3]^ Most benign lesions may only require follow-up or medical treatment, while most malignant lesions require surgical intervention.^[4]^ Therefore, the differential diagnosis between benign and malignant lesions is of significant clinical importance. However, due to the inherent specificity of clinical and biological findings, the diagnosis of orbital lesions remains challenging.^[3–5]^With advancements in medical imaging, ultrasound, CT, and MRI have provided valuable information.^[6]^ MRI, in particular, offers precise anatomical descriptions, as well as differentiation between benign and malignant lesions and qualitative diagnosis for orbital diseases.^[7,8]^ Nevertheless, pathological evidence remains the gold standard for diagnosing orbital diseases. However, tissue extraction usually requires invasive surgery, carrying the risks of surgery and biopsy, such as functional impairment, disfigurement, vision loss, or other severe complications.^[9]^ In such cases, it is essential to find new noninvasive tools to differentiate orbital lesions.

Contrast-enhanced CT imaging, due to its high-resolution image quality and short scan time, has been widely applied in the evaluation of orbital lesions. CT imaging can clearly display tumor size, shape, and infiltration within the orbit, aiding in the initial screening of orbital lesions. However, relying solely on visual analysis still has certain limitations, as a physician’s experience and subjective judgment can affect the accuracy and consistency of the diagnosis. To overcome these challenges, radiomics technology has emerged as a new method that analyzes a large number of quantitative features extracted from medical images. It can objectively and systematically explore the microscopic information hidden in the images, providing more precise diagnostic evidence.^[10,11]^ In recent years, radiomics, which involves extracting quantitative features from images, has been widely applied in early diagnosis, prognostic evaluation, and treatment response prediction of tumors.^[12,13]^ Contrast-enhanced CT radiomics can uncover hidden microscopic features in the images, further improving the accuracy of distinguishing between benign and malignant orbital lesions.

This study aims to explore a method for differentiating benign and malignant orbital lesions based on CT imaging features through radiomic analysis. The findings of this study are expected to provide an efficient and objective auxiliary diagnostic tool for ophthalmologists, improving the early detection and treatment of orbital lesions.^[14]^

## 2. Methods and materials

### 2.1. Patients

**Figure 1.**
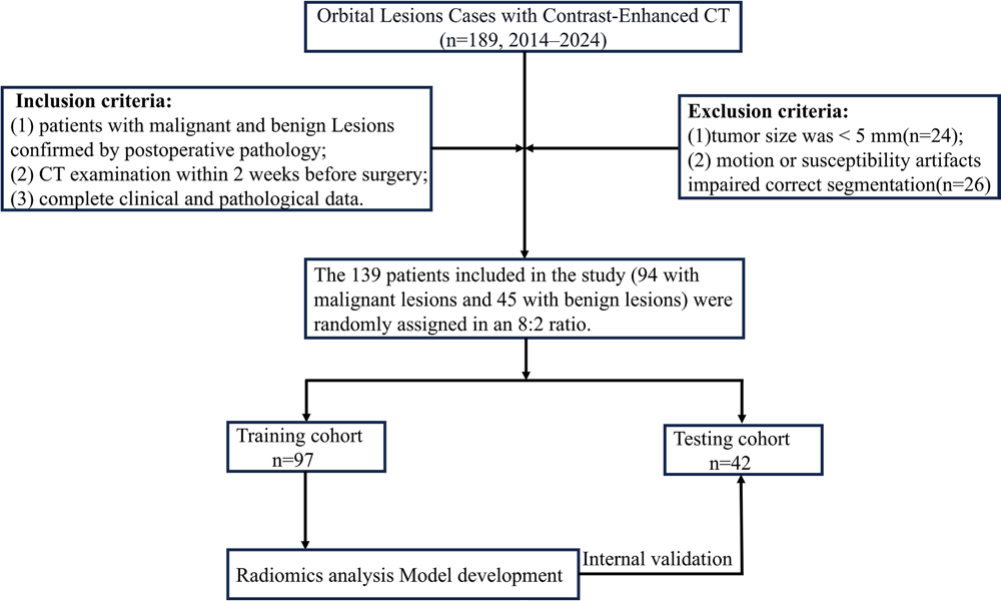
Flowchart of the process of inclusion and exclusion of participants in the study. CT = computed tomography.

**Figure 2.**
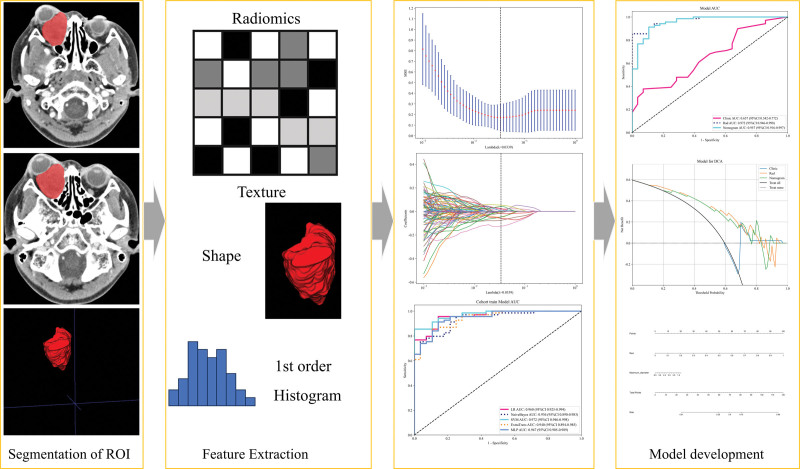
The flowchart of this study. AUC = area under the curve, CI = confidence interval.

This study was approved by the Medical Ethics Committee of Quzhou People’s Hospital, and patients were exempted from the requirement for informed consent (Approval NO: 2024-139). A retrospective analysis was conducted on the contrast-enhanced CT imaging data of 189 patients diagnosed with orbital lesions at our hospital from December 2014 to April 2024. The inclusion criteria were as follows: patients with malignant and benign tumors confirmed by postoperative pathology; CT examination conducted within 2 weeks before surgery; and complete clinical and pathological data. The exclusion criteria were tumor size < 5 mm and motion or susceptibility artifacts that impaired accurate segmentation. Ultimately, 97 patients with malignant tumors and 48 patients with benign tumors were included in the study. Details of patient selection are shown in Figure 1. Figure 2 presents the flowchart of this study, which includes patient collection and grouping, image preprocessing, feature extraction, feature analysis, and model construction. All patients were randomly assigned to the training group and the testing group in an 8:2 ratio.

### 2.2. Clinical baseline characteristics and CT image acquisition

Age and gender data for all patients were collected from the clinical medical records system. CT images were acquired using high-resolution CT scanners (SOMATOM Sensation 16, Siemens Medical Systems, Forchheim, Germany; SOMATOM Definition Edge, Siemens Medical Systems, Forchheim, Germany) and Philips 16-slice multi-detector CT scanner. Scan parameters were as follows: for the first scanner, tube voltage 120 kV, tube current 240 mAs, collimator width 6 mm × 0.75 mm, bed speed 0.75 mm/s; for the second scanner, tube voltage 125 kV, tube current 250 mA, slice thickness 5 mm, matrix 256 × 256, and contrast agent iodixanol (dose 1.5 mL/kg body weight). All images (slice thickness 1.25 mm) were reconstructed using soft tissue windows (window width: 100, window level: 45) and then processed and analyzed based on the obtained soft tissue window images.

### 2.3. Image segmentation

Accurate segmentation of orbital lesions is the prerequisite for image analysis. A radiologist (M.Q.) used the open-source ITK-SNAP software (version 3.6.0; http://www.itk-snap.org) to perform manual segmentation. The 3-dimensional volume of interest was delineated by stacking the region of interest slice-by-slice to cover the entire tumor. To verify interobserver reproducibility, 30 randomly selected lesions were segmented by another radiologist. The intraclass correlation coefficient (ICC) was used to determine the inter-observer agreement of radiomics feature data, where an ICC ≥ 0.8 was deemed to indicate acceptable reliability.

### 2.4. Radiomics feature extraction

To avoid data leakage, feature selection was performed using only training group data. Prior to feature extraction, images were standardized. All images underwent isotropic interpolation to generate isotropic 3D data with pixel spacing of 1mm, which was then uniformly used as input for grayscale feature extraction and filtering transformations. The feature extraction algorithm adhered to the Image Biomarker Standardization Initiative. Radiomics features were extracted using the open-source Python 3.6-based package Pyradiomics (http://pypi.org/project/pyradiomics/). Extracted radiomics features included first-order statistics, shape features, Gray Level Size Zone Matrix, Gray Level Run Length Matrix, Neighborhood Gray Tone Difference Matrix, and Gray Level Dependence Matrix, among others.

### 2.5. Feature selection and fusion

To select radiomics features with good reproducibility and low redundancy, the ICCs between the radiomics features were first calculated. Features with ICCs ≥ 0.8 were selected twice, reducing the number of features from 1834 to 724. Then, for features with high repeatability, Spearman rank correlation coefficient was calculated to express the relationship between features. For feature pairs with correlation coefficients >0.9, one of the features was retained. To maximize the representational ability of features, a greedy algorithm was employed for feature selection, where the feature with the highest redundancy in the current feature cohort was removed in each step. Through Spearman correlation coefficient screening and greedy selection, the number of features was reduced from 724 to 145. Finally, the least absolute shrinkage and selection operator (LASSO) algorithm was used to shrink some regression coefficients to zero by constructing a penalty function λ, thereby incorporating stable radiomics features into the LASSO-Cox analysis. The optimal λ value was determined by 5-fold cross-validation (see File S1, Supplemental Digital Content, https://links.lww.com/MD/Q559 for detailed parameter specifications.), and the non-zero coefficients and corresponding weights of the radiomics parameters were selected based on the optimal λ value.

### 2.6. Model development

After feature fusion and selection, we used the scikit-learn machine learning library to construct multiple classification models. The constructed machine learning classification models included logistic regression (LR), NaiveBayes, support vector machine (SVM), ExtraTrees, and multilayer perceptron (MLP). All models were trained on the training cohort using a grid search algorithm to fine-tune commonly used hyperparameters. To prevent overfitting, 5-fold cross-validation was performed on the training cohort to select the optimal parameters for each classification model. Finally, the optimal radiomics feature importance scores were obtained. The performance of each predictive model was comprehensively evaluated by plotting the receiver operating characteristic (ROC) curve and calculating the area under the curve (AUC), accuracy, sensitivity, and specificity. A nomogram was constructed by combining clinical baseline features to visualize the classification evaluation results.

### 2.7. Statistical analysis

Univariate comparisons (using *t*-tests, the Mann–Whitney *U* test, and/or the chi-square test, depending on the case) were conducted to assess the differences in clinical characteristics and radiomics scores between malignant and benign tumors. To assess the diagnostic performance of both the radiomics model and the readers, ROC curves were generated. The AUC, accuracy, sensitivity, specificity, positive predictive value, and negative predictive value were calculated for all groups. The DeLong test was applied to compare the AUC values of the various models. To evaluate the calibration of the radiomics nomogram, the Hosmer–Lemeshow test was utilized. Decision curve analysis (DCA) was performed to measure the net benefit of the models at varying threshold probabilities. All statistical analyses were executed using R software (version 3.5.2; www.r-project.org), with statistical significance cohort at *P* < .05.

## 3. Results

### 3.1. Selection of clinically significant features

Among the 189 patients who met the inclusion criteria, 50 were excluded due to the following reasons: tumor size was < 0.5 cm (24 cases) and poor image quality or presence of motion artifacts (26 cases). Ultimately, 139 patients were included in the study, consisting of 65 females and 74 males. Table 1 summarizes the baseline characteristics of orbital malignant and benign tumors in the training and testing cohorts. Univariate and multivariate logistic regression analyses (Table 2) showed that none of the clinical factors demonstrated statistically significant associations (*P* > .5). However, a high maximum tumor diameter is a well-established imaging biomarker associated with malignancy, as it reflects the rate of tumor progression.^[15]^ Therefore, the maximum tumor diameter was selected as the sole clinical factor for inclusion in the final nomogram model.

**Table 1:** Baseline patient characteristics in the training cohort and testing cohort.

	Training cohort	*P*-value	Testing cohort	*P*-value
Age (yr)*	44.44 ± 22.18	.414	41.59 ± 23.11	.281
Gender		.545		1.0
Females	41		24	
Males	56		18	
Maximum diameter*	2.78 ± 1.12	.056	2.64 ± 1.13	.778
Minimum diameter*	1.68 ± 0.69	.434	1.63 ± 0.62	.959

* Data are mean ± standard deviation (range).

**Table 2:** Univariate and multivariate logistic regression analysis of clinical risk factors for orbital lesions.

Clinical risk factors	Univariate logistic regression		Multivariate logistic regression	
	OR (95% CI)	*P*	OR (95% CI)	*P*
Age	1.018 (1.010–1.025)	.000	1.002 (0.987–1.017)	.833
Gender	2.111 (1.319–3.380)	.000	0.547 (0.251–1.192)	.203
Maximum diameter	1.422 (1.241–1.631)	.000	1.706 (0.980–2.004)	.113
Minimum diameter	1.722 (1.381–2.145)	.000	0.871 (0.378–2.004)	.785

Data in parentheses are 95% confidence intervals.

CI = confidence interval, OR = odds ratio.

### 3.2. Feature extraction and model development

**Figure 3.**
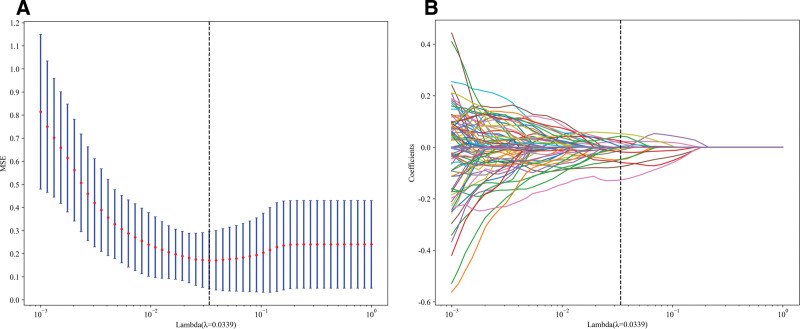
Imaging radiomics feature selection using the least absolute shrinkage and selection operator (LASSO) regression model.

**Figure 4.**
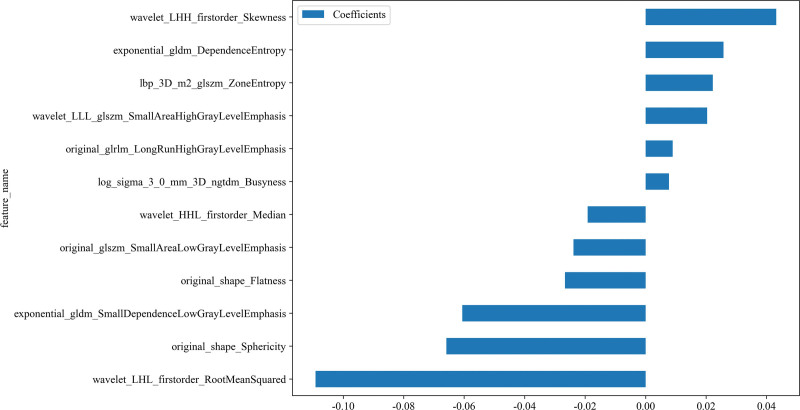
The weight values of the 12 imaging radiomics features were calculated.

**Figure 5.**
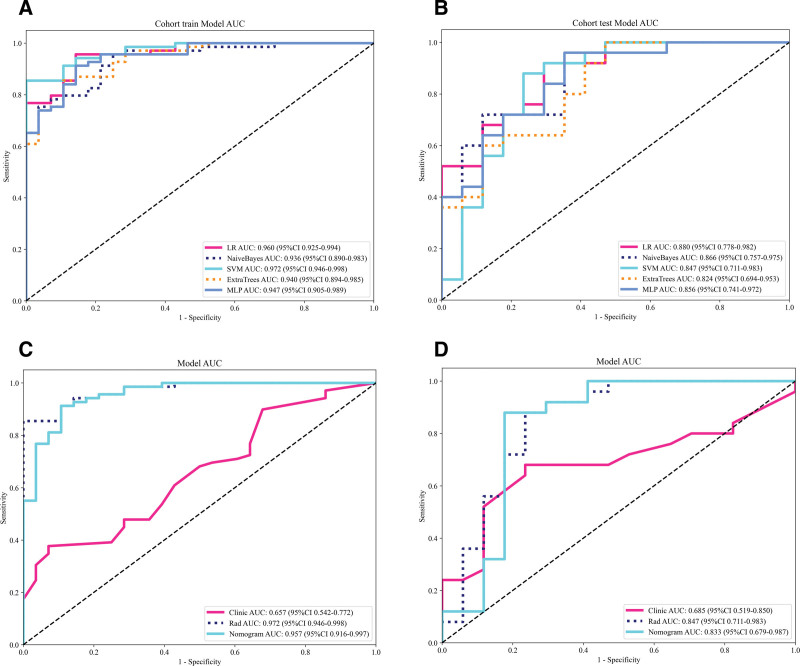
The corresponding ROC curves of the 5 machine learning models. AUC = area under the curve, CI = confidence interval.

A total of 1834 radiomics features were extracted from the CT images of the patients. After conducting variance analysis with 5-fold cross-validation and LASSO selection (Fig. 3A, B), 12 features were retained (Fig. 4), including first-order statistics (3), shape features (2), Gray Level Dependence Matrix (1), Neighborhood Gray Tone Difference Matrix (2), Gray Level Size Zone Matrix (3), and Gray Level Run Length Matrix (1). The data were randomly divided into a training cohort (n = 97) and a Testing cohort (n = 42) at an 8:2 ratio, and 5 machine learning methods were employed to construct the radiomics models (Fig. 5A, B). The results of the models are shown in Table 3. Among them, the SVM model exhibited the best predictive performance in the training cohort, with similar and stable performance in both the training and testing cohorts. Therefore, the SVM model was used to construct the nomogram. Logistic regression was employed to establish the prediction model based on the selected feature parameters, and the radiomics signature (Rad-signature) for each sample was computed to reflect the risk of malignancy. The Rad-signature is the sum of the radiomics features and their corresponding coefficients, as shown in Figure 4.

**Table 3:** Data summary for the 5 models.

Models	Training cohort (n = 97)				Testing cohort (n = 42)			
	AUC (95% CI)	Acc	Sen	Spe	AUC (95% CI)	Acc	Sen	Spe
LR	0.960 (0.925–0.994)	0.928	0.957	0.857	0.880 (0.778–0.982)	0.833	0.920	0.706
NaiveBayes	0.936 (0.890–0.983)	0.814	0.754	0.964	0.866 (0.757–0.975)	0.786	0.720	0.882
SVM	0.972 (0.947–0.998)	0.897	0.855	1.000	0.847 (0.711–0.983)	0.833	0.880	0.765
ExtraTrees	0.940 (0.895–0.985)	0.866	0.855	0.893	0.824 (0.694–0.953)	0.810	1.000	0.529
MLP	0.947 (0.905–0.989)	0.897	0.913	0.857	0.856 (0.741–0.972)	0.833	0.960	0.647

Data in parentheses are 95% confidence intervals. Identical accuracy values across different models reflect identical classification results on the testing cohort, not duplication or error in calculation.

Acc = accuracy, AUC = area under the curve, CI = confidence interval, ExtraTrees = Extra Trees Classifier, LR = logistic regression, NaiveBayes = Naive Bayes Classifier, MLP = multilayer perceptron, Sen = sensitivity, SVM = support vector machine, Spe = specificity.

### 3.3. Nomogram construction and evaluation

**Figure 6.**
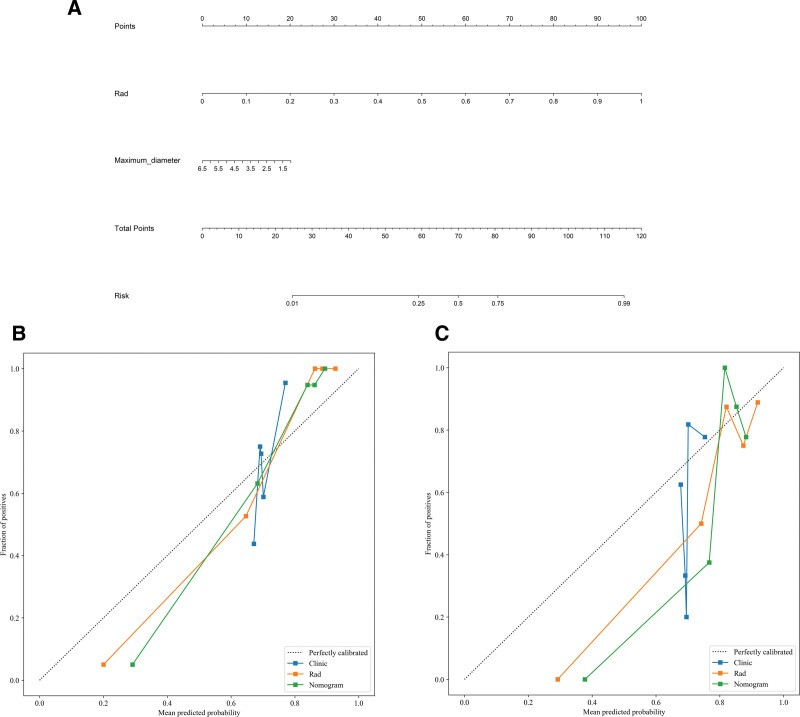
Performance of the Nomogram model.

**Figure 7.**
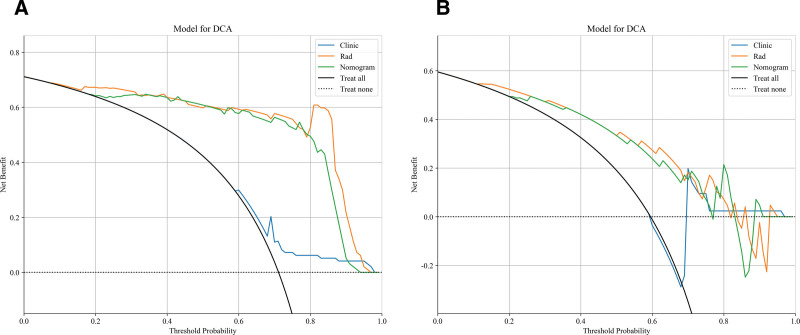
Decision curve analysis of the 3 models in the training cohort (A) and validation cohort (B). DCA = Decision curve analysis.

The maximum tumor diameter and Rad-signature were incorporated into the nomogram model (Fig. 6A). Calibration curves and the Hosmer-Lemeshow test demonstrated good calibration and fit of the nomogram model (Fig. 6B, C). The diagnostic performance of the clinical model, radiomics model, and nomogram model in both the training and testing cohorts is presented in Table 4. The ROC curves for each model are shown in Figure 5C and 5D. DeLong’s test revealed that the diagnostic performance of both the radiomics model and the nomogram model was significantly superior to that of the clinical model (*P* < .05), with no statistically significant difference observed between the radiomics and nomogram models (*P* = .301, .084). DCA showed that both the radiomics and nomogram models provided higher net benefit for distinguishing malignant and benign orbital lesions compared to the clinical model (Fig. 7).

**Table 4:** Diagnostic efficiency of different models in the training cohort and testing cohort.

	AUC (95% CI)	Accuracy	Sensitivity	Specificity	PPV	NPV
Training						
Clinic	0.657 (0.542–0.772)	0.536	0.377	0.929	0.929	0.377
Radiomics	0.972 (0.947–0.998)	0.897	0.855	1.000	1.000	0.737
Nomogram	0.957 (0.916–0.997)	0.907	0.913	0.893	0.955	0.806
Testing						
Clinic	0.685 (0.519–0.850)	0.714	0.680	0.765	0.810	0.619
Radiomics	0.847 (0.711–0.983)	0.833	0.880	0.765	0.846	0.812
Nomogram	0.833 (0.679–0.987)	0.857	0.880	0.824	0.880	0.824

Data in parentheses are 95% confidence intervals. Identical sensitivity values across different models reflect identical classification results on the testing cohort, not duplication or error in calculation.

AUC = area under the curve, CI = confidence interval, NPV = negative predictive value, PPV = positive predictive value.

### 3.4. Application of the nomogram model

**Figure 8.**
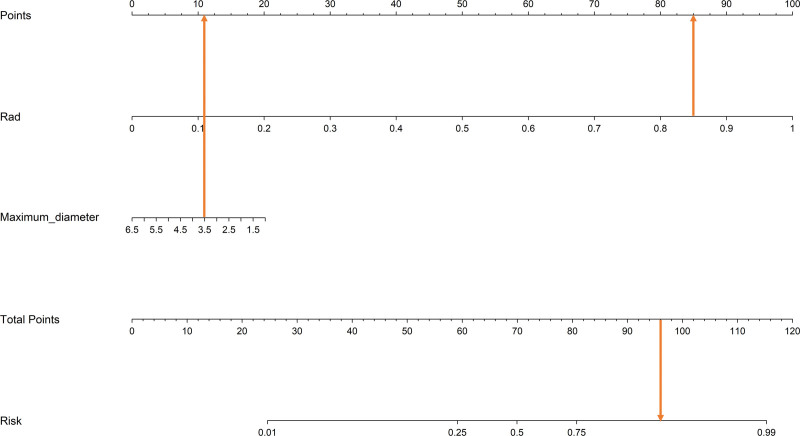
Nomogram score plot for a patient with a malignant tumor in the left orbital cavity.

Figure 8 illustrates the typical clinical application of the nomogram. A 57-year-old male patient was admitted due to right-eye protrusion and restricted movement for half a month. CT imaging revealed a soft tissue mass in the extraconal region of the right orbit, with a maximum diameter of approximately 3.5 cm. The radiomics score was 0.85, and the nomogram score for the lesion was 111, with a malignancy probability > 0.85. Postoperative pathology confirmed that the lesion was an orbital rhabdomyosarcoma, a malignant lesion.

## 4. Discussion

This study aimed to evaluate the potential application of radiomic features derived from enhanced CT imaging in the differentiation between benign and malignant orbital tumors. By performing radiomic analysis on the enhanced CT images of 139 patients with orbital tumors and combining machine learning models, we explored the value of radiomic features in distinguishing benign from malignant orbital lesions. The results indicated that machine learning models based on radiomics, particularly the SVM model, exhibited high predictive performance and robustness in differentiating benign and malignant orbital tumors.

Previous studies have also explored imaging-based approaches for differentiating benign from malignant orbital lesions. For example, Duron et al employed MRI-based radiomics and demonstrated promising diagnostic performance, highlighting the advantage of superior soft-tissue contrast on MRI for lesion characterization.^[4]^ In contrast, our study focuses on CT-based radiomics, which may be more widely applicable given the availability and cost-effectiveness of CT in routine clinical practice, especially as CT is often the first-line imaging modality for orbital evaluation. More recently, end-to-end deep learning models on CT images have also been developed for orbital tumor diagnosis.^[16]^ Compared with radiomics, deep learning has the potential to achieve higher levels of automated performance but generally requires large annotated datasets and is often criticized for its lack of interpretability.^[17,18]^ Radiomics, on the other hand, provides explicit and interpretable quantitative features, which may be advantageous in smaller datasets and can assist clinicians in understanding the basis of model predictions.^[12]^ Taken together, these approaches are complementary, and future work combining radiomics with deep learning strategies, and validating them across multiple centers, may further enhance diagnostic accuracy and clinical applicability.

Firstly, our study confirmed the advantages of radiomics in the diagnosis of orbital tumors. By extracting approximately 1834 radiomic features from enhanced CT images and performing feature selection using methods such as LASSO and mRMR, we ultimately identified 12 optimal features. Compared to traditional imaging diagnostic methods, radiomics can uncover more latent microscopic features, providing more objective and precise lesion information. Various machine learning classification models, including LR, Naive Bayes, SVM, ExtraTrees, and MLP, were evaluated for their performance. The results revealed that the SVM model achieved the best predictive performance, with AUC values of 0.957 in the training cohort and 0.833 in the testing cohort. In this study, the SVM, a common supervised learning method, demonstrated the best predictive performance. SVM effectively handles complex nonlinear problems by searching for an optimal hyperplane in a high-dimensional space, which separates data points of different categories.^[19,20]^ However, the performance drop between the training cohort (AUC 0.972) and the testing cohort (AUC 0.847) suggests potential overfitting. This performance gap may be attributed to several factors, including the relatively small sample size, class imbalance, and the risk of overfitting when training models on limited data. In the differentiation of orbital tumors, SVM leveraged the complex information contained within the radiomic features, thereby improving the model’s prediction accuracy and robustness.^[21]^

SVM exhibited high robustness in our study, especially when handling different datasets, where the model consistently demonstrated stable and accurate performance.^[22]^ By utilizing kernel functions, SVM can construct a more accurate classification boundary in a higher-dimensional feature space, enabling effective differentiation between benign and malignant orbital tumors, even in cases where tumor morphology, size, and boundaries are unclear.^[23,24]^ This feature makes SVM an important tool for solving medical image classification problems, particularly for high-dimensional and complex medical data such as CT images.^[25]^ Additionally, the SVM model’s training process, which involves support vectors (samples that play a key role in determining the decision boundary), makes it less sensitive to noise and outliers, further enhancing the model’s stability and accuracy.^[26,27]^ In this study, the SVM model outperformed other machine learning models in terms of AUC values in both the training and Testing cohorts, indicating its strong generalization ability in distinguishing benign and malignant orbital lesions. This can help clinicians more accurately assess the nature of lesions, thereby improving diagnostic efficiency and treatment outcomes.

Secondly, the construction of a nomogram model further enhanced the ability to distinguish benign from malignant orbital tumors. Based on the SVM model, we combined radiomic features with clinical factors such as the maximum tumor diameter to construct the nomogram model. The AUC value of the nomogram model was significantly higher than that of traditional clinical models, indicating its potential application value in orbital lesion diagnosis. Furthermore, there was no statistically significant difference in diagnostic efficacy between the radiomics model and the nomogram model, suggesting that both models have similar predictive capabilities to some extent. DCA showed that both the radiomics and nomogram models yielded higher net benefits in distinguishing benign and malignant orbital tumors compared to clinical models, further validating the clinical applicability and advantages of radiomics. This indicates that radiomics holds strong clinical potential, particularly for patients who cannot undergo invasive examinations. Although its value has been demonstrated in various ophthalmic diseases, research on orbital lesion analysis remains limited. The few published studies involved small sample sizes and were mainly focused on specific orbital sites or tumor subtypes, such as lacrimal gland tumors, or primarily addressed technical details.^[28–34]^

This study also revealed the promising prospects of radiomics technology in the diagnosis of orbital tumors. While traditional imaging methods such as CT and MRI provide valuable structural information, they often fail to adequately reflect the microscopic features of tumors, especially when tumor boundaries are unclear or in the early stages of disease. Radiomics technology, through the quantitative analysis of hidden microscopic features within images, helps clinicians better classify lesions, particularly in complex cases. Furthermore, by integrating clinical information, radiomics models can provide more comprehensive diagnostic support, improving diagnostic accuracy and consistency.

However, there are several limitations in this study. First, although we used multiple machine learning methods to train and compare models, the analysis was based solely on retrospective data from a single-center study. This reliance on data from a single hospital may introduce selection bias and limit the external validity of the findings. Moreover, the retrospective nature makes the study susceptible to information bias, and the absence of prospective validation further reduces the strength of the conclusions. Second, the final cohort included only 139 patients (94 malignant and 45 benign), resulting in both a relatively small sample size and class imbalance, which may inflate performance metrics and limit generalizability. Third, excluding patients with tumors smaller than 0.5 cm may introduce bias, as small orbital tumors are relatively rare, and the small size of these tumors made it difficult to extract meaningful radiomic features, leading to their exclusion. This may overrepresent larger, more easily detectable tumors. Fourth, although interobserver agreement (ICC ≥ 0.8) was reported for 30 cases, the reproducibility across the entire dataset remains uncertain. The manual segmentation performed by only 2 radiologists may limit the robustness and clinical scalability of the findings. Future studies should incorporate a larger number of radiologists and explore automated or semi-automated segmentation methods to improve reproducibility and scalability. Finally, the nomogram was applied to only one patient as a preliminary demonstration; therefore, larger patient cohorts are required in future work to comprehensively evaluate its reliability and robustness.

## 5. Conclusions

This study demonstrates the potential of enhanced CT radiomics analysis in differentiating benign from malignant orbital tumors. When combined with machine learning methods, it can provide more accurate and objective auxiliary diagnostic tools for clinical practice. As radiomics technology and machine learning models continue to evolve, they are expected to play an increasingly important role in the early diagnosis and treatment decision-making of orbital tumors in the field of ophthalmology.

## Author contributions

**Conceptualization:** Weitao Huang, Xiaohui Liu.

**Data curation:** Weitao Huang, Xiaohui Liu.

**Formal analysis:** Weitao Huang.

**Funding acquisition:** Guozheng Zhang, Xiaohui Liu.

**Investigation:** Weitao Huang.

**Methodology:** Weitao Huang.

**Project administration:** Weitao Huang.

**Visualization:** Guozheng Zhang.

**Writing – original draft:** Weitao Huang, Xiaowei Han, Xiaohui Liu.

**Writing – review & editing:** Xiaowei Han, Guozheng Zhang, Xiaohui Liu.

## Supplementary Material


