# Supplementary material for: Radiomics-based identification of benign and malignant orbital lesions using contrast-enhanced CT imaging
Source: Medicine (Baltimore). 2025 Nov 7;104(45):e45791. doi: 10.1097/MD.0000000000045791 (PMC12599769; doi:10.1097/MD.0000000000045791)
Supplement: Supplementary file 1 [file medi-104-e45791-s001.pdf]

Hyperparameter data adjustment and 5-fold cross-validation explanation:

1.Our hyperparameter tuning was conducted as follows:
LR = LogisticRegression(penalty='none', max_iter=50)
SVM = SVC(probability=True, max_iter=100, kernel='linear')
MLP=MLPClassifier(hidden_layer_sizes=(61,128,64,32),max_iter=100,solver='sgd', random_state=0)
ExtraTrees=ExtraTreesClassifier(n_estimators=60,max_depth=5,min_samples_split=2, random_state=0)

2.The 5-fold cross-validation we performed was as follows: We specified the number of random iterations as 5. In each iteration, 80% of the data was used for training and a random 20% was used for testing, aiming to identify the best model and the corresponding optimal data partitioning. It is important to note that this partitioning was applied exclusively to the training set.
